# Palliative radiation therapy for symptomatic advance breast cancer

**DOI:** 10.1038/s41598-021-84872-9

**Published:** 2021-03-05

**Authors:** Galia Jacobson, Orit Kaidar-Person, Ory Haisraely, Shira Galper, Tatiana Rabin, Ilanit Dromi Shahadi, Yaacov Richard Lawrence, Zvi Symon, Merav Akiva Ben-David

**Affiliations:** 1 Radiation Oncology, Chaim Sheba Medical Center, Ramat Gan, Israel; 2 Sackler School of Medicine, Tel Aviv University, Tel Aviv, Israel; 3 GROW-School for Oncology and Developmental Biology or GROW (Maastro), Maastricht University, Maastricht, The Netherlands; 4 Radiation Oncology, Sourasky Medical Center, Tel Aviv, Israel; 5 Assuta Medical Center, Tel Aviv, Israel

**Keywords:** Breast cancer, Medical research, Oncology

## Abstract

In this study, we evaluated the effectiveness of palliative breast radiation therapy (RT), with single fraction RT compared with fractionated RT. Our study showed that both RT fractionation schemas provide palliation. Single fraction RT allowed for treatment with minimal interference with systemic therapy, whereas fractionated RT provided a more durable palliative response. Due to equivalent palliative response, at our institution we have increasingly been providing single fraction RT palliation during the COVID-19 pandemic.

## Introduction

While breast cancer is usually detected at an early stage, between 10 and 30% of patients present with locally advanced breast cancer (LABC) and 5–10% have distant metastases at initial presentation^1^. About 30% of patients with metastatic breast cancer present with a local or locoregional recurrence. Local recurrence may cause severe symptoms that impact patients' quality of life. Whenever possible, locoregional recurrence is usually treated aggressively with surgery and/or radiation with or without systemic therapy. However, in cases of very advance or metastatic disease it is not always feasible. Advanced breast/chest wall disease present as fungating or ulcerating breast mass with symptoms of pain, mass effect, bleeding, malodorous discharge or subsequent infection^2^.

It has been shown that women with advanced breast disease can suffer from psychologic distress^3^ leading to depression, embarrassment, fear, shame and social isolation^4^. In case of a symptomatic breast tumor, the local disease often requires palliative management to control symptoms and improve quality of life (QOL). Therefore, treatment should aim to controlling these symptoms, in hope that it will may improve patient’s QOL.

Current guidelines recommend that patients with symptomatic breast tumors, especially for patients with systemic disease, should receive systemic therapy rather than therapies directed to their primary tumor^5,6^. Most clinical trials focus on therapeutic efficacy of systemic therapy in the adjuvant setting and systemic response in the metastatic setting^7^ and less reported the palliative effect of such treatments in case of symptomatic breast lesions^8–10^.

Radiation therapy (RT) has an important role in the palliative treatment of ulcerating/fungating breast lesions, but to date, there are no prospective studies that have examined clinical outcomes of palliative RT as they relate to dose and fractionation.

At our institution we treat over 650 new breast cancer patients per year, approximately one percent of whom are referred for palliative RT due to ulcerating or fungating breast lesions.

The purposes of this study are to describe our institutional experience with palliative breast irradiation and evaluate the clinical effectiveness of different radiation dose fractionation protocols.

## Patients and methods

The study was approved by the Institutional (Sheba Medical Center) Review Board (IRB). Patient consent was waived by the IRB due to the retrospective nature of the study. All methods were performed in accordance with the relevant guidelines and regulations.

We conducted a retrospective chart review of consecutive breast cancer patients treated with radiation therapy at our institution between August 2006 and August 2016.

Data collected included patient demographics, tumor characteristics, treatment, and oncological outcomes. Treatment outcomes were assessed by physician observation at follow up visits. Outcomes reported included wound healing, reduction in mass size, pain control and bleeding cessation. Radiation associated acute and late toxicities were recorded using the Common Toxicity Criteria Scale CTCEA v4.03^11^.

Only patients with metastatic breast cancer who had breast lesions with skin invasion or ulceration/mass forming who received palliative RT to the breast and had minimum follow up of 2 months following radiation therapy were included in this study. Data are presented in descriptive statistics.

### Ethical approval

All procedures performed in studies involving human participants were in accordance with the ethical standards of the institutional and/or national research committee and with the 1964 Helsinki declaration and its later amendments or comparable ethical standards.

### Conference presentation

This study was presented at the 2017 ASTRO meeting, San Diego, California.

## Results

Of 4220 patients treated with RT for breast cancer during the study period, a total of 53 patients (1.3%) were included in the final analysis with a mean follow up of 15 months (range 2–94) after RT.

Mean age was 62 years (range 27–92). All patients had stage IV disease at time of palliative breast RT and 32 of the patients had metastatic disease at time of breast cancer diagnosis.

Median time from diagnosis of primary breast cancer to delivery of palliative RT was 23 months (range 0–72). Nine patients (16.9%) had previous breast RT.

Mean breast tumor size was 6.3 cm (range 2–35 cm, median 6 cm). Nine patients (16.8%) had tumors larger than 9 cm.

All patients reported discomfort, 45 patients (84%) reported pain, 23 (43%) had exudative secreting tumor, 16 (30%) had bleeding associated with an ulcerative wound, and 12 patients (22%) had malodor. 32 patients (60%) had two or more symptoms at presentation. Patient and tumor characteristics are presented in Table 1.

**Table 1:** Patients and tumor characteristics.

Parameter	n
Patients	53
Age at radiation, mean (year), (range)	62 (27–92)
**Histology**	
IDC	36 (68%)
ILC	3 (5.6%)
Other/unknown	14 (26.4%)
**Hormonal status**	
ER positive	40 (75.4%)
PR positive	15 (28.3%)
HER2 positive	17 (32%)
Triple negative	5 (9%)
**Stage at diagnosis**	
Local	21 (40%)
Metastatic	32 (60%)
Previous adjuvant breast radiation	9 (16.9%)
Time from diagnosis to palliative radiation, median (m), (range)	23 (0–72)
**Tumor characteristic**	
**Size**	
**Mean**	6.3 cm
< 4 cm	6 (11%)
4–9 cm	31 (58.2%)
> 9 cm	9 (16.9%)
Unknown	7 (13.2%)
Skin involvement	32 (60%)
**Symptoms**^**a**^	
Discomfort	53 (100%)
Pain	45 (84%)
Ulceration	26 (49%)
Discharge	23 (43%)
Bleeding	16 (30%)
Malodor	12 (23%)
Chemotherapy during palliative radiation	13 (24%)

^a^Some patients had more than one symptom.

Forty-six patients were treated with photon radiation and seven patients were treated using electrons (9–15 meV) to the breast or chest wall. All patients were treated using a 1 cm bolus over the treatment field.

Per our department protocol: 9 patients received 8 Gy in a single fraction, and 44 patients received fractionated regimens. Fractionated regimens included: 45 Gy in 15 fractions, 39 Gy in 13 fractions, and 50 Gy in 25 fractions.

### Single fraction radiation

For the nine patients receiving a single fraction treatment, mean age was 75 years and all had poor performance status (ECOG 3–4). Two patient received chest wall RT, and seven received breast RT. None of the patients had previous breast RT. The common indications for referring to RT were massive bleeding in five patients and severe pain in 4 patients.

Seven patients (78%) experienced a clinical benefit: 3/4 (75%) reported decrease in pain level and reduction in narcotics consumption and 4/5 (80%) had no recurrent bleeding. The only reported RT toxicity was grade 1 dermatitis in all patients.

Treatment outcomes for single fraction and fractionated radiation are presented in Table 2. Four patients (44%) required re-irradiation and received another single fraction of 8 Gy, median time to re-irradiation was 2 months (range 0.5–9 months) after initial RT single fraction. Reasons for re-irradiation were recurrent bleeding (in 3 out of 4) or persistent pain (in 1 out of 4). All four patients had clinical benefit with symptomatic improvement after the second fraction.

**Table 2:** Single versus fractionated protocol outcome.

	Fractionated treatment	Single fraction
Clinical benefit	44/44 (100%)	7/9 (77%)
Re-irradiation	8 /44 (18%)	4/9 (44%)
Time to re-irradiation (mean)	16 months	3 months

### Fractionated radiation

**Figure 1:**
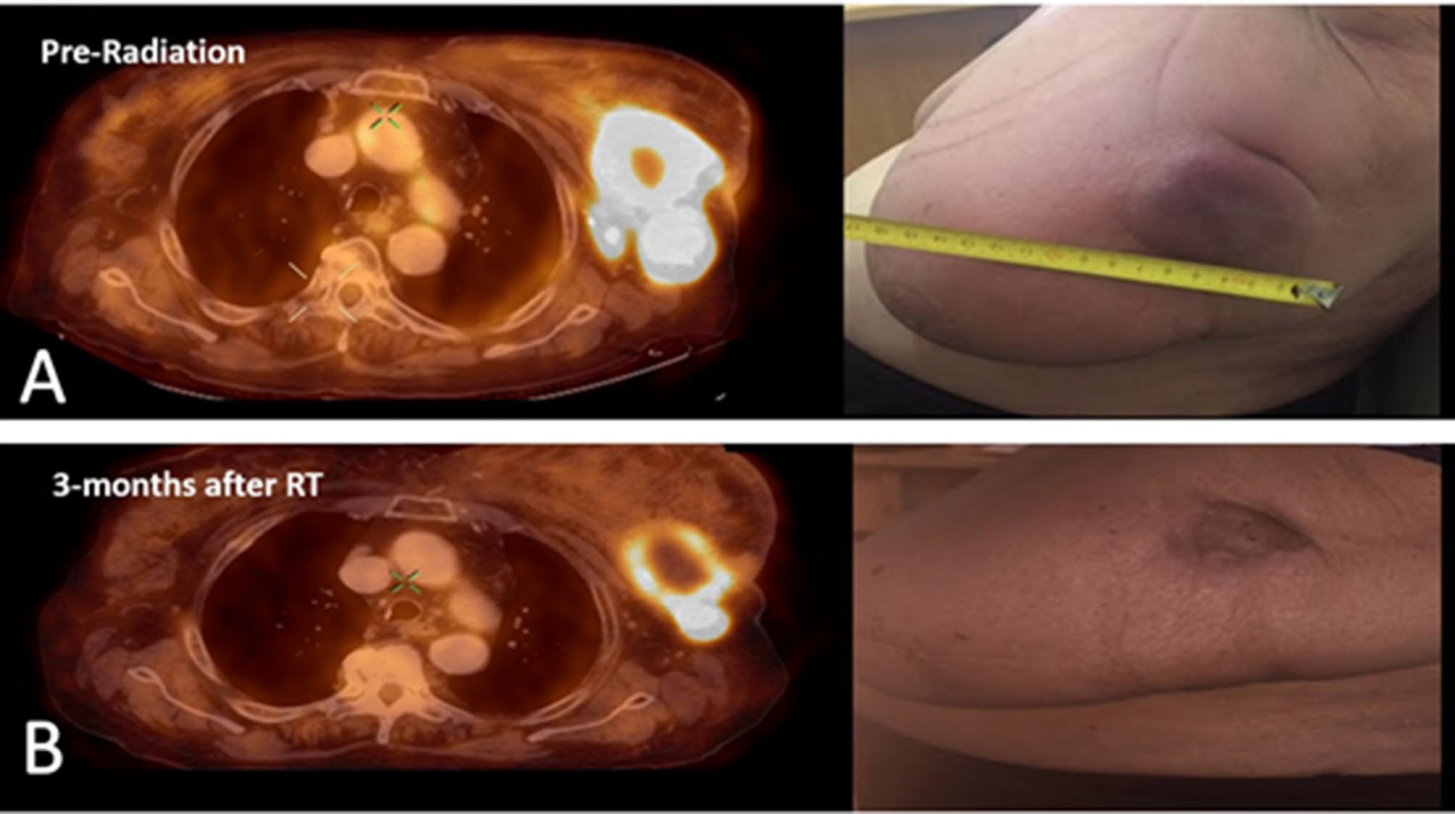
A PET-CT scan and clinical evaluation of a patient with locally advanced breast cancer who was treated with fractionated radiation for palliation. (**A**) Pre-radiation therapy, (**B**) 3 months after radiation therapy.

Forty-four patients received fractionated RT (Fig. 1). Mean age was 61 years. Nine patients had previously received adjuvant RT at time of primary diagnosis: 77% previously received a total dose of 50 Gy in 25 fractions and 23% received a total dose of 42.4 Gy in 16 fractions. Mean time from primary adjuvant radiation to palliative radiation was 65 months (23–182). The indications for palliative radiation were pain (52%), bleeding (22%), discharge (19%), or a combination of symptoms (7%). Median total dose of palliative radiation RT was 39 Gy. Forty-three patients (97%) completed the full prescribed dose.

All patients reported a clinical benefit: 10/11 patients (91%) reported bleeding cassation, 14/18 patients (78%) reported decrease in wound discharge, 26/36 patients (72%) reported a decrease in pain level and narcotic consumption, and 6/8 (75%) reported a decrease in malodor. Of note, all patients with malodorous discharge received oral metronidazole before or during RT.

Eight patients (18%) required re-irradiation with a median time to re-irradiation of 13.5 months (range 1–36 months). All patients eventually had systemic disease progression and forty patients (91%) had further systemic therapy, sixteen patients (36%) had stable/complete long-term response, greater than 10 months, in their irradiated breast despite systemic progression.

RT toxicity included grade 1–2 dermatitis in 90% of the patients. One patient developed recall dermatitis (following treatment with chemotherapy combination of carboplatin-docetaxel 2 weeks following the end of radiation), and two patients had grade 3 radiation pneumonitis that resolved after short course of oral steroids.

## Discussion

Current guidelines for palliative RT for symptomatic breast tumors suggest several RT fractionation schemas based on patient prognosis and performance status. These include a single fraction of 8–10 Gy or 20 Gy in 4–5 fractions for patients with poor prognosis or poor performance status, and 30 Gy in 10 fractions or 50 Gy in 25 fractions for patients with average- good prognosis and good performance status^5,10^. However, due to the paucity of literature on dose fractionation in breast palliation, the optimal radiation dose fractionation for effective treatment of fungating breast lesions is unknown. In the current study we report our experience of palliative RT for treating fungating or ulcerating breast tumors.

In our series, palliative RT was an effective treatment for pain, discomfort, ulceration, bleeding, and malodor. A durable response was seen with either single fraction or multi-fraction RT for most patients. Toxicity was tolerable, with mostly grade 1–2 normal tissue complications. Prior adjuvant RT to the same breast did not appear to impact toxicity and should not be a contraindication for palliative breast re-irradiation. This concurs with the report by Vempati et al., describing no excessive side effects in re- irradiation of previously irradiated breasts. This is probably due to the long interval between the first (adjuvant) radiation and palliative RT, a median of 65 months in our study^12^.

Vempati et al. observed a dose–response effect in their series: six of the nine patients who received 30 Gy or more reported a clinical benefit, whereas none of the four patients who received less than 30 Gy experienced any benefit. In our series we did not detect a threshold dose for clinical benefit or the need for re-irradiation. There were no differences between patients who received below or above the median dose of 39 Gy (EQD2 46.6 Gy) or a dose of 30 Gy (EQD2 34.5 Gy). Other series describe diverse, non-uniform treatment protocols: doses, fractionation, ± hyperthermia^13–15^. In our cohort, single fraction RT was inferior to fractionated RT in terms of clinical benefit and need for re-irradiation. However, those receiving a single fraction were older and had a lower performance status and worse prognosis. Single fraction palliative RT may be less clinically effective than fractionated RT, but it was still beneficial and may be the optimal treatment for this poorer performance status cohort of patients. Single fraction RT has the additional advantage of not requiring a treatment interruption of systemic therapy and it often is less of a burden on patients and their families than fractionated therapy due to fewer treatment visits. A repeat single RT fraction can be safely be administered as needed.

The benefit of loco-regional treatment to the breast by means of surgery and/or radiation in women with metastatic disease is controversial. Some studies have suggested that aggressive local treatment with surgical resection or RT in the metastatic setting is associated with improved survival^7,16–18^. Le Scodan et al. observed a survival improvement with loco-regional treatment, in particular RT, to the breast, among patients with synchronous metastasis. In their study, tumor-stage (T stage) was not associated with improved outcomes and there are no details about breast associated symptoms^19^. In our patient population, 90% of the patients in the fractionated treatment group received systemic therapy following RT, and 36% had long term local control in their irradiated breast.

A potential disadvantage of fractionated RT is delay in systemic treatment. While the long-term effects of delaying systemic treatment in metastatic disease for a few weeks can be debated, women with symptoms stemming from their primary tumor can receive durable long-term benefit from effective palliative breast RT prior to systemic therapy with minimal side effects.

In the single fraction group, all patient received 8 Gy. Higher dose for single fraction RT was reviewed in the adjuvant setting in a single institution retrospective cohort, using 16 Gy fraction brachytherapy in elderly low risk breast cancer patient, with promising safety results^20^.

Future effort can be directed to confirm efficacy and safety of a higher single fraction dose when compared with 8 Gy.

Our study has several limitations including its retrospective nature, small sample size, different RT fractionations and relatively short follow up. We did not use objective measures to assess response and did not take into consideration tumor subtype or systemic therapy (number of treatment lines or type of systemic therapy). While a definitive conclusion regarding RT fractionation cannot be generated from this study, our experience along with the published literature suggest that single fraction RT with 8 Gy can provide good palliation for patients with poor prognosis or poor performance status with ulcerative breast lesions. However, fractionated treatment with a minimal dose of 26–30 Gy may be required to achieve a more durable and efficacious response. In general, our standard regimen for RT palliation is 30 Gy in 10 fractions for patient with good-average performance status and 8 Gy for those with poor performance status. Currently, due to the COVID-19 pandemic many RT protocols have been changes worldwide, including for breast cancer^21^. At time of the COVID-19  pandemic we are increasingly using a single 8 Gy fraction even for those with a better performance status and re-evaluate the need for an additional single dose at one-month follow-up^21^. This allows for adequate palliation, fewer hospital visits and the potential for re-treatment as needed.

## Conclusions

RT can provide durable palliation, reduce pain and bleeding with minimal and tolerable toxicity, even if given as a single RT fraction. However, fractionated therapy provides a more durable response. Prospective studies combining new systemic treatments with RT are needed to improve palliative care of locally uncontrolled breast cancer.
